# Cereblon gene variants and clinical outcome in multiple myeloma patients treated with lenalidomide

**DOI:** 10.1038/s41598-019-51446-9

**Published:** 2019-10-16

**Authors:** Phoebe A. Huang, Shaunna L. Beedie, Cindy H. Chau, David J. Venzon, Sheryl Gere, Dickran Kazandjian, Neha Korde, Sham Mailankody, Ola Landgren, William D. Figg

**Affiliations:** 1 Genitourinary Malignancies Branch, Center for Cancer Research, National Cancer Institute, Bethesda, MD USA; 2 Biostatistics and Data Management Section, National Cancer Institute, Bethesda, MD USA; 3 Myeloma Program, Lymphoid Malignancies Branch, Center for Cancer Research, National Cancer Institute, Bethesda, MD USA; 4 Myeloma Service, Department of Medicine, Memorial Sloan Kettering Cancer Center, New York, NY USA

**Keywords:** Targeted therapies, Myeloma, Genetic markers, Predictive markers

## Abstract

Carfilzomib-lenalidomide-dexamethasone (KRd) therapy has yielded promising results in patients with newly diagnosed multiple myeloma (NDMM). Cereblon (CRBN) is the direct molecular target of lenalidomide and genetic polymorphisms in *CRBN* have been associated with lenalidomide efficacy. In this study, we assessed the correlation of five single nucleotide variants (SNVs) in the *CRBN* gene with clinical response and outcomes in patients with NDMM administered KRd therapy with lenalidomide maintenance, achieving favorable trial endpoints in a prospective Phase II study (NCT01402284). Of the observed SNVs, no associations with KRd therapy response were found in this patient cohort, although strong trends in hypoalbuminemia grade and hyperbilirubinemia grade emerged across the *CRBN* rs1672753 genotype (P = 0.0008) and the rs1714327 genotype (P = 0.0010), respectively. Our results do not provide conclusive support for the predictive utility of *CRBN* gene polymorphisms as potential biomarkers of clinical response to lenalidomide-based therapy in our patient population. However, these findings remain to be validated in prospective studies using larger patient populations.

## Introduction

Thalidomide and its derivatives, the immunomodulatory drugs (IMiDs) lenalidomide and pomalidomide, are used to treat several hematological malignancies, including multiple myeloma (MM)^1,2^. Although only 30% of patients respond to IMiDs used as single agents^3^, triplet combination therapies involving a proteasome inhibitor (carfilzomib), immunomodulatory agent (lenalidomide), and a corticosteroid (dexamethasone) are clinically effective, yielding complete or deep responses in patients with newly diagnosed multiple myeloma (NDMM)^4,5^ and relapsed or refractory MM^6^. Carfilzomib-lenalidomide-dexamethasone (KRd) therapy has resulted in improved progression-free survival (PFS) over administration of lenalidomide and dexamethasone alone^2,5,6^.

The anti-myeloma activity of IMiDs has been attributed to several mechanisms of action, including anti-angiogenic, pro-apoptotic, and anti-proliferative effects^1,7^. Until recently, however, the precise molecular mechanisms by which thalidomide and its analogs act remained elusive. A body of preclinical evidence now exists showing that cereblon (CRBN), a ubiquitously expressed E3 ligase protein, is the direct molecular target of IMiDs^8,9^ and its presence is indispensable for IMiD activity^3^. First identified in patients with non-syndromic mental retardation^10^, CRBN has since been studied *in vitro* in MM, myelodysplastic syndrome, and lymphoma cell lines^9^, *in vivo* using zebrafish, chick, and rodent animal models^8,11–14^, and in pre- and post-IMiD treatment tissue samples collected from patients with MM^3^. Upon binding to cereblon, IMiDs induce CRBN-dependent proteasomal degradation and inhibition of IKZF1/3, B cell-specific transcription factors required for both myeloma cell viability and activation of the immune system^1,2,7^.

Recent studies have established a correlation between CRBN expression levels and clinical response to IMiD treatment. High expression of CRBN in patients with NDMM continuing on daily thalidomide maintenance for 2 years was associated with longer PFS and treatment response (P = 0.005)^15^, and has also been shown to enhance the effects of lenalidomide therapy and potentially overcome resistance to treatment^3,16–18^. Conversely, reduced CRBN expression levels have been linked to the development of lenalidomide resistance in human myeloma cells^3^ as well as poor clinical outcomes in patients with either MM^3^ or lower risk myelodysplastic syndrome^16^. Diminished CRBN protein levels was specifically associated with the development of lenalidomide resistance over the course of treatment in 77% of lenalidomide-refractory MM patients, although baseline CRBN expression at diagnosis did not affect overall survival (OS)^19^. In another study of 53 refractory MM patients treated with pomalidomide, CRBN levels were predictive of decreased response rates and significant differences in both PFS (P < 0.001) and OS (P = 0.01)^20^.

Whether these associations may be driven by genetic variations in the *CRBN* gene remains unknown, as there are currently no clear biomarkers that predict response to lenalidomide therapy. Attempts to quantify cereblon’s utility as a clinical biomarker for IMiDs is ongoing^20–23^. One study found an increased prevalence of mutations in both CRBN and the CRBN pathway impacting CRBN-IMiD interactions in patients with multidrug refractory disease, and subsequently observed conferred lenalidomide resistance *in vitro* following the functional introduction of these mutations in MM cells^24^. A recent analysis of acquired pomalidomide-resistance in MM cell lines similarly revealed a range of *CRBN* mutations and CRBN protein loss associated with treatment resistance^25^. Given the documented association between CRBN expression and IMiD treatment response in patients with MM, some studies have also begun to explore single nucleotide variants (SNVs) in the *CRBN* gene as potentially useful biomarkers for the clinical assessment of antimyeloma efficacy, or patient selection for predicted responders before initiating therapy. In a cohort of 144 MM patients compared to 237 matched healthy individuals, two SNVs (rs711613C > T and rs1045433C > T) within the non-coding regions of the *CRBN* gene (intron 1 and 3′-untranslated region, respectively), thought to control CRBN expression, correlated with major differences in MM susceptibility, progression, and response to treatment^26^. Carriers of the rs711613 major allele demonstrated better response to thalidomide treatment (P = 0.023), while the rs1045433 minor allele was found to be more common, but not statistically significant, in patients with complete or partial response after thalidomide treatment (P = 0.092).

The role of *CRBN* genetic variations as biomarkers that may predict clinical response to IMiD-based therapy remains controversial due to inconsistent findings. Two studies examining a SNV located at -29 nucleotides of the 5′-untranslated region (5′UTR) (rs1672753 C > T) yielded contradictory results on the predominance of each allele in myelodysplastic patients, as compared to healthy controls^16,27^. More recently, this SNV was found to have a significant impact on survival outcomes in patients with MM, conferring extended PFS (P = 0.005) and OS (P = 0.023) in patients with the variant genotypes compared to those with two major alleles, independent of thalidomide therapy^28^. Another study consisting of 68 thalidomide-treated patients with MM conversely identified the major allele to be associated with significantly shorter PFS (P = 0.0321), without significantly impacting OS^29^. In another cohort of 169 patients with refractory or relapsed MM treated with lenalidomide regimens, minor allele carriers of two other naturally occurring SNVs (rs1714327C > G and rs1705814C > T) were associated with worse clinical response and shorter PFS (OR = 2.49, P = 0.0054)^30^. Therefore, whether *CRBN* genetic variations can be prognostic markers of myeloma cell biology or predictive biomarkers of clinical response to IMiD-based therapy remain to be determined.

We previously reported results from a prospective Phase II study that included patients with NDMM treated with 8 cycles of KRd therapy, followed by two years of lenalidomide maintenance (KRd-R)^5^. In this patient cohort, this therapy regimen was found to be highly tolerable and demonstrated high rates of MRD negativity, translating into 12-month longer PFS (P < 0.001). The five-year followup to this study demonstrated long term survival benefits, with KRd-R treatment leading to a rapid, deep, and durable overall response rate and sustained MRD-negative complete responses (CRs)^31^. Given cereblon’s central role as a direct molecular target of thalidomide and its analogs, we sought to determine whether *CRBN* genetic variants may predict for the impressive clinical response observed for this patient population on KRd-R therapy. In the current study, the same patient cohort was genotyped for five *CRBN* polymorphisms (rs1714327 C > G, rs1672753 C > T, rs1045433 C > T, rs1705814 C > T, rs711613 C > T). To our knowledge, this study comprises the largest array of *CRBN* SNVs studied for associations with favorable clinical outcomes following KRd-R therapy among a single patient cohort. We assessed for correlations between these *CRBN* SNVs and clinical progression, response to treatment, and toxicity.

## Results and Discussion

We aimed to assess the prognostic significance of previously described SNVs in the *CRBN* gene, which may affect the expression, activity, or alternative splicing of its protein, in a single cohort of patients with NDMM demonstrating impressive survival benefits following lenalidomide therapy. We chose a patient population demonstrating promising clinical outcomes, with an overall response rate of 100%^5^, and previously reported differences in mutational patterns between patients with early and later stage disease^32^. Five SNVs in *CRBN* (rs1714327 C > G, n = 42; rs1672753 C > T, n = 40; rs1045433 C > T, n = 43; rs1705814 C > T, n = 44; rs711613 C > T, n = 43) were detected, although low frequencies of homozygous genotypes and the number of determined SNVs limited the power of statistical analyses. The distribution of the *CRBN* genotypes was not significantly correlated with patient demographics, disease markers or baseline laboratory findings (data not shown). Of the observed SNVs, none of the variants appeared to be associated with KRd-R therapy response and/or minimum residual disease status in this patient cohort (Table 1). All patients carrying homozygous wild-type alleles of two SNVs (rs1672753 CC, rs1705814 CC) were MRD-negative (3/3, 7/7), compared to 77% (27/35) and 71% (25/35) of patients carrying variant alleles. Additionally, none of the observed genotypes appeared to be related to progression-free survival (Table 2). Assessing for genotype versus toxicities, all toxicities with sufficient data were tested for associations with *CRBN* SNVs (Supplementary Table 1). Strong trends in hypoalbuminemia grade and hyperbilirubinemia grade emerged across the *CRBN* rs1672753 genotype (P = 0.0008) and the rs1714327 genotype (P = 0.0010), respectively (Table 3). Analysis using Somers’ D statistic indicates that the associations are strong with the rs1672753 variant genotypes having higher hypoalbuminemia grade (D = 0.53) and with the rs1714327 wild-type genotype having higher hyperbilirubinemia grade (D = −0.45) (Supplementary Table 2). The significance of these associations and their relevance as potential biomarkers remain to be determined.

**Table 1:** *CRBN* genotypes versus response and minimum residual disease status.

Response										MRD		
Genotype	sCR	nCR/CR	VGPR	PR/SD	P (trend)^a^	OR (95% CI)	P^b^	OR (95% CI)	P^b^	MRD negative	MRD positive	P (trend)^c^
	*n* (%)	*n* (%)	*n* (%)	*n* (%)		<sCR		<CR		*n* (%)	*n* (%)	
***CRBN rs1714327***												
CC	7 (41)	3 (18)	5 (29)	2(12)	0.55	Ref.		Ref.		12 (75)	4 (25)	0.80
CG	11 (65)	0 (0)	5 (29)	1 (6)		0.38 (0.08, 1.85)	0.30	0.78 (0.16, 3.84)	1.0	13 (76)	4 (24)	
GG	4 (50)	1 (12)	2 (25)	1 (12)		0.70 (0.09, 5.27)	1.0	0.86 (0.10, 6.35)	1.0	6 (86)	1 (14)	
***CRBN rs1672753***												
CC	2 (67)	0 (0)	1 (33)	0 (0)	0.49	Ref.		Ref.		3 (100)	0 (0)	0.56
CT	6 (60)	1 (10)	3 (30)	0 (0)		1.33 (0.05, 98)	1.0	0.86 (0.03, 67.2)	1.0	7 (78)	2 (22)	
TT	14 (52)	3 (11)	7 (26)	3 (11)		1.86 (0.09, 118)	1.0	1.18 (0.05, 76.3)	1.0	20 (77)	6 (23)	
***CRBN rs1045433***												
CC	—	—	—	—	0.54					—	—	0.14
CT	3 (43)	0 (0)	3 (43)	1 (14)		Ref.		Ref.		3 (50)	3 (50)	
TT	20 (56)	3 (8)	9 (25)	4 (11)		0.60 (0.08, 4.17)	0.69	0.42 (0.05, 3.00)	0.4	28 (80)	7 (20)	
***CRBN rs1705814***												
CC	5 (62)	1 (12)	2 (25)	0 (0)	0.33	Ref.		Ref.		7 (100)	0 (0)	0.3
CT	12 (57)	0 (0)	6 (29)	3 (14)		1.25 (0.18, 10.2)	1.0	2.25 (0.29, 27.2)	0.67	15 (71)	6 (29)	
TT	6 (40)	3 (20)	4 (27)	2 (13)		2.50 (0.32, 21.8)	0.40	2.00 (0.23, 26.1)	0.66	10 (71)	4 (29)	
***CRBN rs711613***												
CC	8 (47)	3 (18)	4 (24)	2 (12)	0.55	Ref.		Ref.		12 (75)	4 (25)	0.46
CT	10 (56)	0 (0)	5 (28)	3 (17)		0.71 (0.15, 3.26)	0.74	1.47 (0.31, 7.13)	0.73	12 (67)	6 (33)	
TT	5 (62)	1 (12)	2 (25)	0 (0)		0.53 (0.06, 3.93)	0.67	0.61 (0.05, 5.17)	1.0	7 (100)	0 (0)	

^a^Jonckheer-Terpstra test; ^b^Fisher’s exact test; ^c^Cochran-Amitage test.

Abbreviations: CRBN, cereblon; sCR, stringent complete response; nCR, near complete response; CR, complete response; VGPR, very good partial response; PR, partial response; SD, stable disease; OR, odds ratio; CI, confidence interval; MRD, minimal residual disease.

**Table 2:** *CRBN* genotypes versus progression-free survival on KRd.

Genotype		P (trend); HR (95% CI)	P (log-rank); HR (95% CI)
*CRBN rs1714327*	(CC, CG, GG)	0.15; 0.32 (0.04, 2.7)	0.27; 0.34 (0.07, 1.7)
*CRBN rs1672753*	(CC + CT, TT)		0.14; 4.3 (0.53, 35)
*CRBN rs1045433*	(CC + CT, TT)		0.56; 1.8 (0.23, 15)
*CRBN rs1705814*	(CC, CT, TT)	0.20; 3.2 (0.37, 28)	0.40; 1.6 (0.18, 15)
*CRBN rs711613*	(CC, CT, TT)	0.16; 0.30 (0.04, 2.5)	0.32; 0.44 (0.11, 1.8)

Abbreviations: CRBN, cereblon; KRd, Carfilzomib-lenalidomide-dexamethasone; HR, hazard ratio; CI, confidence interval.

**Table 3:** *CRBN* genotypes versus toxicity grades.

**Hypoalbuminemia Grade**					
Genotype	0	1	2	3	P (trend)
	n (%)	n (%)	n (%)	n (%)	
***CRBN rs1672753***					
CC	1 (33)	2 (67)	0 (0)	0 (0)	0.0008
CT	2 (20)	8 (80)	0 (0)	0 (0)	
TT	1 (4)	13 (48)	10 (37)	3 (11)	
**Hyperbilirubinemia Grade**					
	0	1	2		P (trend)
***CRBN rs1714327***					
CC	3 (18)	8 (47)	6 (35)		0.0010
CG	11 (65)	4 (24)	2 (12)		
GG	6 (75)	2 (25)	0 (0)		

The discovery of cereblon as the direct target of thalidomide and its derivatives has significantly heightened interest in its potential use as a biomarker of clinical response and outcome^3,15,33,34^. Cereblon is a necessary component of the cullin ring E3 ubiquitin ligase complex required for lenalidomide efficacy^15,34^ and specific *CRBN* variants are thought to be potential genetic markers of clinical response or outcome following KRd therapy in patients with MM^26^. To date, the role of CRBN as a biomarker for treatment response and/or resistance has not been strongly established, and these findings on the association of *CRBN* SNVs with patient outcomes remain controversial^21,24,35,36^. For example, a recent analysis of CRBN gene expression levels in patients enrolled in STRATUS, a Phase IIIb study evaluating the safety and efficacy of pomalidomide treatment, found no notable difference in overall response rate in high versus low CRBN expressers^37^, despite previously documented associations between CRBN expression and IMiD treatment response in patients with MM^18,20,38^.

Due to insufficient data on *CRBN* gene variants and their impact on clinical response to therapy, larger scale studies are needed to determine the prognostic significance of *CRBN* SNVs. The reported incidence of direct polymorphisms in *CRBN* and in publicly available MM sequencing data is low, except in single case reports or *in vitro* cell line studies^24,36,39^. A similar genotyping study on the effects of a *CRBN* coding region SNV (rs121918368 C > T) only detected the wild-type allele in the genotyped patient cohort^34^, while another study failed to identify any CRBN or CRBN pathway variants in all samples analyzed from a cohort of 21 patients with MM^35^. Likewise, our study was limited by a small sample size in patient subgroups and few progressions, thus the results should be viewed cautiously. Discrepancies reported in previous studies indicate a need for larger patient cohorts to confirm our findings, which warrant future replication studies with expanded study groups to examine the potential predictive utility of *CRBN* SNVs as biomarkers^16,21,27,36^.

Future genotyping studies would further benefit from a thorough functional analysis of the investigated *CRBN* genetic variants to elucidate the biological mechanisms underlying any potential associations with response to lenalidomide therapy. Three of the substitutions chosen (rs711613 C > T, rs1045433 C > T, and rs1672753 C > T) are located in non-coding regions of *CRBN* (intron 1, 3′UTR, and 5′UTR, respectively), which may be associated with lenalidomide efficacy^26^. However, the exact functional consequences of the SNVs investigated in this study are currently unknown. Potential mechanisms underlying the effects of cereblon include modulation of its gene and protein expression or alternative splicing, particularly via removal of exon 10 containing the IMiD-binding domain^15,16,21^.

A molecular analysis of cereblon-related resistance to IMiD therapy in a longitudinal study of 1000 patients with NDMM from multiple clinical sites revealed a subset of IMiD-treated patients exhibiting significant reductions in CRBN expression and copy number loss at relapse compared to baseline levels, whereas patients with copy number gains at baseline appeared to benefit from IMiD therapy^40^. Given the central role of cereblon as a substrate receptor within the E3 ubiquitin ligase complex, which contains other proteins required to carry out the ubiquitination functions regulating MM cell survival, the substrate specificity and/or ability of cereblon to recognize or bind to its partners may additionally be determined by *CRBN* genetic variants^10^. Other studies have proposed alternative mechanisms including epigenetic, transcriptional, and/or post-transcriptional modulation of *CRBN* gene expression that may drive clinical response to lenalidomide therapy^20,36,41^. In addition, mutational screening of NDMM patients receiving lenalidomide in the Phase II GEM10MAS65 trial implicated several downstream genes in the CRBN pathway (such as IKZF1, IKZF2, IRF4, and MYC) as other potential biomarkers of lenalidomide resistance^42^. Molecular studies have also implicated alternative binding targets^43,44^ and mechanisms of action^8,14,45^, which may be cell-type specific, and (by extension) disease-specific, which may explain the variability in reported correlations between CRBN expression and patient response to IMiDs. Although CRBN is known to be the direct target of lenalidomide and its presence is essential to IMiD activity, whether IMiDs may target other molecules downstream of CRBN and/or different signaling pathways resulting in treatment resistance has not been well established^3^.

We previously reported results from a clinical study with positive trial endpoints using the same cohort of lenalidomide-treated NDMM patients^5^. Our group identified putative *VEGF* and *VEGFR2* SNVs that may be possible markers of clinical response following KRd combination therapy^46^. Moreover, we recently identified a significant mutational burden in our patient population of NDMM of recurrent genetic mutations implicated in MM^32^. The current exploratory genotyping study was the first, to the best of our knowledge, to evaluate the association of multiple *CRBN* genetic variations with clinical response and outcome within a patient population demonstrating impressive survival benefits, including an 100% overall response rate. While we did not find any significant correlations of these five *CRBN* variants with either treatment response or progression-free survival in our limited patient population, whether *CRBN* polymorphisms have a role as predictive biomarkers of response and/or outcome needs to be verified in other MM patients on KRd therapy. Future genetic and functional studies of these causal variants are warranted to confirm the findings observed from this patient population. An improved understanding of the molecular consequences of *CRBN* polymorphisms may aid the development of personalized treatment regimens for MM and address whether these SNVs may serve as promising biomarkers for lenalidomide-based therapy.

## Methods

### Patients

Patients (*n* = 45) with newly diagnosed multiple myeloma (NDMM) were enrolled in a prospective Phase II study (NCT01402284) and received eight 28-day cycles of KRd followed by lenalidomide maintenance for 2 years after completing 8 cycles of the combination therapy. Details regarding patient characteristics, response criteria, minimum residual diseases (MRD) monitoring methods, dosing regimens, and pharmacokinetics data have been previously reported^47^. The studies were approved by the Institutional Review Board at the National Cancer Institute, and all participants provided written informed consent. All research methods were performed in accordance with relevant guidelines and regulations.

### Genotyping

A QiaBlood DNA extraction kit was used to extract DNA from samples of whole blood, as per the manufacturer’s instructions (Qiagen, Valencia, CA). Primer pairs were designed or used as previously published^27^ to amplify and determine *CRBN* polymorphisms using a nested PCR protocol. Big Dye Terminator Cycle Sequencing Ready Reaction kit V1.1 was used to perform direct nucleotide sequencing PCR on an ABI Prism 310 Genetic Analyzer (Applied BioSystems, Foster City, CA). Quality of the amplified PCR products was verified by agarose gel electrophoresis for each sample tested. The primer sequences for each SNV are listed in Supplementary Table 3. The genotype analysis was performed independently by two persons and repeat sequence analysis was performed to confirm all individuals expressing the variant genotypes.

### Statistical considerations

Trends in continuous distributions and dichotomous factors across three genotypes were assessed using the Jonckheere-Terpstra and Cochran-Armitage tests, respectively, while the Wilcoxon rank sum test and Fisher’s exact test were used for comparisons of two genotypes or groups. Associations between continuous and ordered categorical variables were quantified using the Spearman rank correlation coefficient (*r*) and Somers’ D statistic, while the Chi squared test was used to ascertain Hardy-Weinberg equilibrium and consistency with previously published genotype frequencies. Progression-free survival was measured from the on-study date to the date of progression or last follow-up. Genotypes and categorical variables were compared using the log-rank test, and hazard ratios were estimated using proportional hazards regression. Exact tests were used as appropriate to calculate *P* values and 95% confidence intervals (CIs). Large sample CIs of test statistics may be inconsistent with exact *P* values in cases where exact CI methods are not available in the software employed (SAS/STAT 12.1). The *P* values reported are not corrected for multiple comparisons.

## Supplementary information

Supplementary Tables
