# Supplementary material for: Cereblon gene variants and clinical outcome in multiple myeloma patients treated with lenalidomide
Source: Sci Rep. 2019 Oct 16;9:14884. doi: 10.1038/s41598-019-51446-9 (PMC6795854; doi:10.1038/s41598-019-51446-9)
Supplement: Supplementary file 1 — Supplementary Tables [file 41598_2019_51446_MOESM1_ESM.docx]

**SREP-19-13642TB** - Supplementary Information

**Cereblon gene variants and clinical outcome in multiple myeloma patients treated with lenalidomide**

Phoebe A. Huang^1^, Shaunna L. Beedie^1^, Cindy H. Chau^1^, David J. Venzon^2^, Sheryl Gere^1^, Dickran Kazandjian^3^, Neha Korde^4^, Sham Mailankody^4^, Ola Landgren^4^, and William D. Figg^1,*^

^1^Genitourinary Malignancies Branch, Center for Cancer Research, National Cancer Institute, Bethesda, MD; ^2^Biostatistics and Data Management Section, National Cancer Institute, Bethesda, MD; ^3^Myeloma Program, Lymphoid Malignancies Branch, Center for Cancer Research, National Cancer Institute, Bethesda, MD; and ^4^Myeloma Service, Department of Medicine, Memorial Sloan Kettering Cancer Center, New York, NY, USA

**Supplementary Table 1. Association of *CRBN* genotypes versus toxicities (P-trend)**

|  |  |  | P (trend) |  |  |
| --- | --- | --- | --- | --- | --- |
| Toxicity | rs1714327 | rs1672753 | rs1045433 | rs1705814 | rs711613 |
| LYMPHOPENIA | 0.11 | 0.047 | 0.81 | 0.14 | 0.14 |
| THROMBOCYTOPENIA | 0.91 | 0.12 | 0.47 | 0.73 | 0.90 |
| LEUKOPENIA | 0.76 | 0.10 | 0.97 | 0.87 | 0.90 |
| ANEMIA | 0.26 | 0.60 | 0.17 | 0.34 | 0.44 |
| NEUTROPENIA | 0.086 | 0.036 | 0.50 | 0.23 | 0.20 |
| ALT INCREASE | 0.42 | 0.21 | 0.56 | 0.78 | 0.54 |
| AST INCREASE | 0.75 | 1.00 | 0.70 | 0.61 | 0.55 |
| ALKALINE PHOSPHATASE | 0.70 | 0.14 | 0.93 | 0.72 | 0.91 |
| HYPOALBUMINEMIA | 0.10 | 0.0008 | 0.11 | 0.12 | 0.035 |
| HYPERBILIRUBINEMIA | 0.0010 | 0.40 | 0.35 | 0.019 | 0.0047 |
| CREATININE INCREASE | 0.83 | 0.038 | 0.26 | 0.86 | 0.90 |
| PERIPHERAL NEUROPATHY | 0.88 | 0.082 | 0.59 | 0.83 | 0.93 |
| DIARRHEA | 0.34 | 0.78 | 1.00 | 0.094 | 0.44 |
| CONSTIPATION | 0.92 | 0.82 | 0.78 | 0.87 | 0.82 |
| EDEMA | 0.82 | 0.65 | 0.14 | 0.94 | 0.96 |
| INJECTION SITE REACTION | 0.36 | 0.31 | 0.23 | 0.64 | 0.71 |
| RASH MACULOPAPULAR | 0.81 | 0.51 | 0.89 | 0.67 | 0.82 |
| HYPERMAGNESIMIA | 0.19 | 0.47 | 0.70 | 0.33 | 0.30 |
| HYPERNATREMIA | 0.81 | 0.79 | 0.68 | 0.69 | 0.93 |
| HYPOCALCEMIA | 0.99 | 0.62 | 1.00 | 0.99 | 0.42 |
| HYPOMAGNESEMIA | 0.48 | 0.60 | 1.00 | 0.47 | 0.83 |
| HYPOPHOSPHATEMIA | 0.83 | 0.72 | 0.76 | 0.90 | 0.96 |
| PAIN | 0.29 | 0.10 | 0.37 | 0.49 | 0.83 |
| FATIGUE | 0.87 | 0.97 | 0.66 | 0.82 | 0.96 |
| INSOMNIA | 0.46 | 0.043 | 0.74 | 0.69 | 0.31 |
| DYSPNEA | 0.69 | 0.53 | 0.43 | 0.97 | 0.59 |
| UPPER RESPIRATORY INFECTION | 0.50 | 1.00 | 1.00 | 0.65 | 0.41 |

**Supplementary Table 2. Association of *CRBN* genotype versus toxicities (Somers’ D)**

|  |  |  | Somers' D |  |  |
| --- | --- | --- | --- | --- | --- |
| Toxicity | rs1714327 | rs1672753 | rs1045433 | rs1705814 | rs711613 |
| LYMPHOPENIA | -0.22 | 0.31 | -0.06 | 0.20 | -0.20 |
| THROMBOCYTOPENIA | -0.02 | 0.26 | 0.16 | -0.05 | -0.02 |
| LEUKOPENIA | -0.05 | 0.29 | 0.02 | -0.02 | 0.02 |
| ANEMIA | -0.17 | 0.10 | 0.33 | 0.14 | -0.11 |
| NEUTROPENIA | -0.25 | 0.38 | 0.17 | 0.18 | -0.19 |
| ALT INCREASE | -0.11 | 0.21 | 0.14 | 0.04 | -0.08 |
| AST INCREASE | 0.04 | 0.00 | 0.11 | -0.07 | 0.08 |
| ALKALINE PHOSPHATASE | -0.05 | 0.23 | -0.04 | -0.05 | 0.02 |
| HYPOALBUMINEMIA | -0.22 | 0.53 | 0.37 | 0.20 | -0.28 |
| HYPERBILIRUBINEMIA | -0.45 | 0.14 | 0.23 | 0.32 | -0.38 |
| CREATININE INCREASE | 0.03 | 0.33 | 0.25 | -0.02 | -0.02 |
| PERIPHERAL NEUROPATHY | -0.03 | 0.29 | 0.14 | 0.03 | -0.01 |
| DIARRHEA | -0.13 | 0.05 | 0.00 | 0.23 | -0.11 |
| CONSTIPATION | -0.01 | -0.04 | -0.08 | 0.02 | 0.03 |
| EDEMA | -0.03 | 0.08 | 0.35 | -0.01 | -0.01 |
| INJECTION SITE REACTION | -0.13 | 0.17 | 0.28 | 0.06 | -0.05 |
| RASH MACULOPAPULAR | -0.04 | 0.12 | -0.03 | 0.06 | -0.03 |
| HYPERMAGNESIMIA | -0.18 | -0.13 | 0.10 | 0.14 | -0.14 |
| HYPERNATREMIA | -0.04 | 0.04 | 0.15 | 0.05 | 0.01 |
| HYPOCALCEMIA | 0.00 | 0.08 | 0.00 | 0.00 | 0.11 |
| HYPOMAGNESEMIA | 0.09 | 0.09 | -0.04 | -0.09 | 0.03 |
| HYPOPHOSPHATEMIA | -0.03 | 0.07 | -0.10 | -0.02 | -0.01 |
| PAIN | -0.14 | 0.27 | 0.18 | 0.09 | -0.03 |
| FATIGUE | -0.03 | 0.01 | 0.09 | 0.03 | -0.01 |
| INSOMNIA | -0.10 | 0.34 | 0.09 | 0.05 | -0.14 |
| DYSPNEA | -0.06 | 0.11 | 0.19 | -0.01 | -0.08 |
| UPPER RESPIRATORY INFECTION | -0.08 | 0.01 | -0.05 | 0.07 | -0.11 |

| **Supplementary Table 3. *CRBN* primers used in this study** | |  |
| --- | --- | --- |
|  |  |  |
|  |  |  |
|  | Forward Primer | Reverse Primer |
|  |  |  |
|  |  |  |
| **rs1714327C>G** | F1-GAAGAGTGACTTGTACACAGAA | R1-ACTGGCTTTGGTGAAAAACCA |
|  | F2-CACAGAGAATAGTGGGATTA | R2-GGACAAAAGCATTTTTCACC |
|  | F3-CATTACTGAATGGGGGGTGGATCT | R3-ATCCAGAAATGCTTTGTCACTAGG |
| **rs1672753C>T** | F1-TGTACGCCGGGCACTCAGCCGTG | R1-GCAACAGAGCAGCGAAGAAA |
|  | F2-GCAGGCCTGTAATTGTCCC | R2-AAGCAGCTTTCCGGTGCGGCCCTG |
|  | F3-TCCCGCCTAGGCCATCACTTTCAG | R3-GTGGTTGCCCATGTTGTGC |
| **rs1045433C>T** | F1-GTCTGGTTTTGTCATGTGTAA | R1-CTACTACTATAGGGAAAAGATGCC |
|  | F2-GAGAGGTACTATGCTACTGACAGG | R2-CCTCACCCAGACTAAACTTGA |
|  | F3-GGAACAAACCAGACCAGTGAAGTC | R3-GCTGCTTTGCCACCTACCTCTT |
| **rs1705814C>T** | F1-AGAGTAGTCAAGCATCTCTCAC | R1-CCAGATCCATCAGAGGAATC |
|  | F2- AGCTGAAAGAGGCCAAACGC | R2-TCCTGGTCACAGAATGG |
|  | F3-CAGGGGCTAATAGCTGGTGACT | R3-CTGGGTGACCATGTACATTGTC |
| **rs711613C>T** | F1-CCAGCAACTTAGACCCAATATAC | R1-CCGATTTGATAAGCGAGTATTG |
|  | F2-GATGTCTCCTGATCTACCTGAA | R2-TAGTTCAATGACTTGCCCAAG |
|  | F3-CCTCTGAAGAGCCACAACAGCATT | R3-CGGTCTTTCTCAGGCATGTACGAT |
|  |  |  |
|  |  |  |
